# *MLC1* alteration in human iPSCs give rise to disease-like cellular vacuolation phenotype in the astrocyte lineage

**DOI:** 10.1186/s13023-026-04316-3

**Published:** 2026-03-26

**Authors:** Saumya Sharma, Vishal Bharti, Prosad Kumar Das, Abdul Rahman, Harshita Sharma, Riya Rauthan, Madhumita RC, Neerja Gupta, Rashmi Shukla, Sujata Mohanty, Madhulika Kabra, Kevin R. Francis, Debojyoti Chakraborty

**Affiliations:** 1 CSIR-Institute of Genomics and Integrative Biology, New Delhi, 110025 India; 2 Academy of Scientific and Innovative Research (AcSIR), Ghaziabad, 201002 India; 3 All India Institute of Medical Sciences (AIIMS), New Delhi, 110029 India; 4 Cellular Therapies and Stem Cell Biology Group, Sanford Research, Sioux Falls, SD 57104 USA; 5 Department of Pediatrics, Sanford School of Medicine, University of South Dakota, Sioux Falls SD, 57105 USA; 6 Hannover Medical School, 30625 Hannover , Germany; 7 Johns Hopkins University School of Medicine, Baltimore , 21205, USA

**Keywords:** Somatic cell reprogramming, CRISPR-Cas9 system, Directed differentiation

## Abstract

**Background:**

Megalencephalic **L**eukoencephalopathy with subcortical **c**ysts (MLC), a rare and progressive neurodegenerative disorder involving the white matter, is not adequately recapitulated by current disease models. Somatic cell reprogramming, along with advancements in genome engineering, will allow the establishment of *in-vitro* human models of MLC for disease modeling and drug screening. In this study, we utilized cellular reprogramming and gene-editing techniques to develop **i**nduced **P**luripotent **S**tem **C**ell (iPSC) models of MLC to recapitulate the cellular context of the classical MLC-impacted nervous system.

**Results:**

MLC iPSCs were comprehensively characterized for pluripotency and were subsequently differentiated to disease-relevant cell types: **N**eural **S**tem **C**ells (NSCs) and astrocytes. RNA (**R**ibo**n**ucleic **a**cid) sequencing profiling of MLC NSCs revealed a set of differentially expressed genes related to neurological disorders and epilepsy, a common clinical finding within MLC disease. This gene set can serve as a target for drug screening for the development of a potential therapeutic for this disease. Upon differentiation to the more disease relevant cell type- astrocytes, MLC-characteristic vacuoles were clearly observed, which were distinctly absent from controls. This emergence recapitulated a distinguishing phenotypic marker of the disease.

**Conclusions:**

Through the creation and analyses of iPSC models of MLC, our work addresses a critical lacunae in the field- relevant cellular models of MLC for use in both disease modeling and drug screening assays. Further investigation can utilize these MLC iPSC models, as well as generated transcriptomic data sets and analyses, to identify potential therapeutic interventions for this debilitating disease.

**Supplementary Information:**

The online version contains supplementary material available at 10.1186/s13023-026-04316-3.

## Background

**M**egalencephalic **L**eukoencephalopathy with subcortical **C**ysts (MLC) is a slowly progressive degenerative brain disease involving the white matter which is a result of a spectrum of pathogenic variants across *MLC1* or *GlialCAM* (**Glial c**ell **a**dhesion **m**olecule) genes. This disease was first discovered independently by Dr. Marjo Van der Knaap in the Netherlands [1], and by Dr. Bhim Sen Singhal in the Indian Agrawal community [2]. Therefore, MLC is also named as Van der Knaap-Singhal disease [3]. The three major categories of causal variations are: autosomal recessive mutation in *MLC1*, autosomal recessive and an autosomal dominant mutation in *GlialCAM* [4]. *MLC1* was the first gene known to cause MLC and was mapped to chromosome 22qtel [5, 6]. *MLC1* translates into a protein MLC1, that is mainly expressed in astrocytes within the brain, especially at astrocytic end feet that contact the blood-brain barrier [7], within the pia mater, and within astrocytes present at the synaptic cleft [8]. The structural features and observed brain defects in patients of MLC, such as brain oedema, fluid filled cysts, vacuolation in astrocytes and hypomyelination, suggest that MLC1 may regulate ionic and water balance within the brain [9–13]. However, a fully elucidated role of MLC1 protein is yet to be deciphered. In MLC, patients develop macrocephaly from an early age with other characteristics such as developmental delay, intellectual disability, motor deficits, seizures, mental decline, and ataxia. Though severity is variable, it is usually a slowly progressive disease and individuals may live into their fifties [14]. MLC is found prevalent in the Turkish [1, 15] and Indian Aggarwal [2, 16–18] communities with some cases found in Japan [19], Israeli community [20], and communities around Finland [5], Italy [21], Egypt [22], and Korea [23] as well.

At present, a cure for MLC is not available and patients rely on supportive treatments. Current management of MLC disease is essentially based on the specific set of symptoms observed in the patients and requires execution of multidisciplinary approaches working towards the goal of seizure control, physical therapy, and avoiding head trauma to the patient (which can temporarily worsen the symptoms) [24]. These management strategies only work as palliative cure and require an absolute therapeutic solution for MLC disease in its repertoire. Since it is a rare genetic brain disease with approximately 200 reported cases worldwide, research towards discovering a therapeutic intervention is needed. Thus, a relevant disease model is needed to identify potential therapies for this debilitating disease.

To understand MLC pathology, various *in-vitro* and *in-vivo* models have been developed. Expressing mutant MLC1 protein in heterologous cell lines (such as HeLa, HEK293 cells and Xenopus oocytes) indicated that missense variations in *MLC1* are more often pathological. They hinder plasma membrane localization of MLC1 protein [25]. Some mutations also affect the half-life of MLC1 protein by causing protein degradation: through protein misfolding and activation of **e**ndoplasmic **r**eticulum **a**ssociated **d**egradation (ERAD) pathway [26]. MLC1 protein is highly expressed in astrocytes; therefore, astrocytes represent a relevant *in-vitro* model system to study MLC1 pathological effects [27]. The U251 human astrocytoma cell line, that expresses almost undetectable levels of endogenous MLC1 protein, was made to stably express *MLC1* gene or *MLC1* gene with several missense/pathological variations. It was discovered that not every mutant protein is able to reach the plasma membrane and might be retained in the endoplasmic reticulum (ER) [9]. Many pathogenic variations altered interaction of MLC1 with its bonafide protein interactors, such as GlialCAM, Kir4.1 (**I**nward **r**ectier type potassium (**K**^+^) channel protein **4.1**), Na K-ATPase (Sodium (**Na**^+^) Potassium (**K**^+^) **A**denosine **T**ri**p**hosphat**ase**), V-ATPase (**V**acuolar-**A**denosine **T**ri**P**hosphat**ase**), and TRPV4 (**T**ransient **r**eceptor **p**otential cation channel subfamily **V** member **4**) [12]. This showed that *in-vitro* modeling of *MLC1* patient variations could specifically reveal variation-specific dysfunctionality; these models can then be used to develop patient-specific therapeutics. *MLC1* suppression in primary rat astrocytes, with small interfering RNA (siRNA) and *MLC1* deletion in mice [10] resulted in emergence of intracellular vacuoles, a pathological hallmark of the MLC affected brain [28]. However, this system did not recapitulate the mis-localization of MLC1 interacting protein ZO-1 as seen in patients. Other mouse models have been generated with knockdown of MLC1 across all tissues [10, 11]. *MLC1* mouse models recapitulate some aspects of human MLC: early onset of disease, increased brain water content, cysts in the white matter, and abnormal astrocytes near the blood brain barrier. Additionally, a zebrafish model has also been developed by deleting the *MLC1* ortholog [29]. However, disease severity in all of these models do not fully recapitulate the actual human condition, with the zebrafish model bearing even less disease. Poor disease recapitulation may be due to differences between species or due to unknown compensatory mechanisms in these model organisms that are possibly absent from the human subjects. In primates, the glia population is more complex and the glia to neuron ratio is also significantly higher [30]. These findings limit the utility of studying MLC in currently available animal models.

With advances in somatic cell reprogramming [31] and CRISPR (**C**lustered **R**egularly **I**nterspaced **S**hort **P**alindromic **R**epeats)- Cas9 (**C**RISPR **as**sociated protein **9**) genome editing [32, 33], multiple new avenues were opened for rare disease research. Neural differentiation and patterning of derived induced pluripotent stem cells (iPSC) can model a diseased brain in a dish in an indirect manner, without causing harm to the donor [34]. Temporal exposure of iPSCs to important signaling molecules has given rise to protocols that can be used to generate distinct populations of neurons and glia from iPSCs [35], including astrocytes. Astrocytes are the primary cell type within the mammalian brain [36]. Astrocytes help neurons by guiding their development and parallelly support them in terms of nutrition and metabolism [37]. They have also been associated with multiple brain pathologies, where defects in their functionality can impact neuronal survival and neurologic function [38]. Astrocytes express certain surface molecules and release factors that affect recovery of the brain after an injury, outgrowth of neurites, synaptic plasticity or neuron regeneration [39]. To understand MLC, which has a direct link with astrocyte activity, it is essential to analyze MLC impacts on disease-relevant neural cell types such as astrocytes to identify disrupted signaling pathways and cellular deficits within MLC.

## Methods

## PBMC isolation and reprogramming to iPSCs

10 ml of freshly drawn blood (from a male patient of MLC) was diluted with PBS (**P**hosphate **b**uffer **s**aline) (1:1) and the dilution is layered over Ficoll Paque (GE 17–1440-02) (Day −4; 4 days prior to the day of reprogramming, day 0). This was followed by centrifugation (330 g, 45 min at 21 °C, 1 unit acceleration and 0 unit deceleration) based separation, to obtain **p**eripheral **b**lood **m**ononuclear **c**ells (PBMCs) at the “buffy coat”. Extracted out the contents of the buffy coat and washed them twice in 5% FBS + 1x DPBS (**D**ulbecco’s **p**hosphate **b**uffer **s**aline) (330 g, 15–30 min at 21 °C, 9 units acceleration and deceleration). Part of the freshly isolated PBMCs were frozen down and the other part, cultured in PBMC media [StemPro®-34 SFM (**S**erum **f**ree **m**edium) Complete Medium (Gibco™) supplemented with L-Glutamine (2 mM)] with cytokines [FLT3 (**F**eline McDonough Sarcoma- **l**ike **t**yrosine kinase **3**; 100 ng/ml; Cat. No.: PHC9415), SCF (**S**tem **c**ell **f**actor;100 ng/ml; Cat. No.: PHC2116), IL-3 (**I**nter**l**eukin-3; 20 ng/ml; Cat. No.: PHC0034), and IL-6 (**I**nter**l**eukin-6; 20 ng/ml; Cat. No.: PHC0065)] for 4 days with daily, half media changes (designated day −4, day −3, day −2 & day −1). On day 0, for reprogramming PBMCs to iPSCs, cells were collected by centrifugation (200 g, 5 min at 21°C) and treated with Sendai virus-based vectors for transduction [40], as per the manufacturer’s instructions given for CytoTune-iPSC 2.0 Sendai Reprogramming kit. On the next day (day 1), cells were collected, centrifuged, and then rinsed with PBMC media to remove viral vectors from spent media. Cells were transferred to an ultra-low attachment plate and incubated for two days without any media changes. On day 3, cells were collected and plated on a fresh, Matrigel-coated, 6 well plate (20,000 - 50,000 cells per well) in PBMC media without cytokines and incubated without disturbing until day 6. On day 6, the entire spent PBMC media was replaced with fresh PBMC media. Transitioning to mTesr1 media began with half media replacement from day 7, followed by a complete media replacement and replenishment from day 8 to day 21 while observing cells for morphological changes. After day 21, ESC (**E**mbryonic **s**tem **c**ell)-like cells were isolated by manual picking of live stained Tra-1–60 positive colonies and transferring these individually to a Matrigel-coated, 24 well plate. These harvested colonies were qualified to be prospective iPSC clones, subjected to further characterizations, allowing them to amplify and propagate onto Matrigel-coated plates and passaging (at the ratio of 1:4) with ReLeSR (**R**eagent for **e**nzyme-free **L**ifting of **e**mbryonic **S**tem cell **R**egions) (Table S3).

## Generation of *MLC1* iPSC models with CRISPR-Cas9 knockout

A single cell suspension was made of NL5 iPSCs [NCRM-5 (RRID:CVCL_1E75)- a kind gift from the National Heart, Lung, and Blood Institute iPSC core facility, NIH, Bethesda, MD; Sex: Male], with the help of accutase. 300,000 cells were counted and centrifuged to a pellet. For each cell pellet, pSpCas9(BB)-2A-GFP (PX458; a kind gift from Feng Zhang, RRID: Addgene_48138) with two guide RNAs targeting exon 1 and exon 2 of *MLC1* gene was taken and a cell + plasmid DNA suspension was made in 15 μl of Buffer R (Neon Electroporation system, Invitrogen). Parallelly, the same dual guide plasmid but with scrambled sequence of crRNA was used as control. Electroporation was carried out by dipping the gold coated tip carrying 10 ul of the suspension into 3 ml of Buffer E (Neon electroporation system, Invitrogen); electroporation parameters were: 1200 V, 30 ms, 1 pulse. The tip contents were thereafter released into a single well of a matrigel coated 24 well plate, now containing STEMacs iPS Brew (Miltenyi Biotec) plus CloneR (Stem Cell Technologies) reagent. The cells were then allowed to repair and replenish for the next 24 hrs, after which a media change was performed. 48 hrs post-electroporation, the cells were collected and a flow cytometry based single cell sorting was performed into a 96 well plate, coated with Matrigel, and containing stem cell media plus CloneR. Sorted cells were allowed to grow until proper colonies were visible and individual cell clones could thereafter be propagated, cryostored, and characterized for pluripotency.

## Characterization of reprogrammed iPSCs and *MLC1* knockout iPSCs

Tra-1–60 immunocytochemistry: Cells were collected in PBS with 5% BSA (**B**ovine **S**erum **a**lbumin). DyLight 488-conjugated Tra-1–60 antibody [(1:100); Cat no.: MA1-023-D488X; Invitrogen; RRID: AB_2536700] was added to cells, incubated on ice for 45 mins, washed 3x with PBS, strained through a 40 μm membrane, and analyzed by FACS (**F**luorescence **a**ctivated **c**ell **s**orting) to assess efficiency of pluripotency attainment.

For pluripotency determination: iPSC clones were subjected to alkaline phosphatase assay as per manufacturer protocol. Patient derived iPSCs were stained with Tra-1–60 antibody. A panel of antibodies were used to assess pluripotency (Table S3).

Trilineage differentiation of iPSCs: Patient-derived iPSCs were subjected to trilineage differentiation using StemDiff trilineage differentiation kit [Cat no.: 05231-Ectoderm; 05232-Mesoderm; 05233-Endoderm; Stem Cell Technologies] following manufacturer’s instructions. For *MLC1* knockout iPSCs and scrambled controls, an undirected differentiation protocol was used. **E**mbryoid **b**odies (EBs) were prepared by forced aggregation of 200–250 cells in an Aggrewell 800 plate in iPSC media without bFGF (**b**asic **f**ibroblast **g**rowth **f**actor) for 6 days. After 6 days, the EBs were plated to 0.1% gelatin-covered dishes in DMEM-F12 (**D**ulbecco’s **M**odified **E**agle **M**edium/Nutrient Mixture **F-12**) media with 20% FBS (**F**etal **B**ovine **S**erum) for 14 days. Trilineage marker profiling was done by performing a qPCR (**q**uantitative **P**olymerase **C**hain **R**eaction) assay with TaqMan probe based qPCR conditions (Primer & oligo list in Table S1 & Table S2).

Immunofluorescence (IF) staining: The expression of pluripotency and tri-lineage markers was studied using IF staining. Cells were fixed in 4% **p**ara**f**orm**a**ldehyde (PFA) for 15 min at room temperature and after PBS washes, incubated in blocking buffer [10% **n**ormal **g**oat **s**erum (NGS), 1% BSA and 0.3% Triton X-100 in PBS] for 1 hr at room temperature. Afterwards, cells were incubated with primary antibodies diluted in their respective dilution buffers [(for trilineage differentiation assay: Sox1 (**S**RY-B**ox** Transcription Factor **1;** SRY (**S**ex-determining **r**egion **Y)**) (in 1% BSA, 1% NGS, 0.25% Triton-X in PBS), Alpha-SMA (Alpha Smooth Muscle Actin) (in blocking buffer), GATA4 (**GATA** Binding Protein **4**) (in 1% BSA and 0.1% Tween20)] and for pluripotency assay: Oct4 (**oct**amer-binding transcription factor **4**), Sox2 (**S**RY-B**ox** Transcription Factor **2**) and Klf4 (**K**rüppel-**l**ike **f**actor **4**)] at 4 °C overnight and then incubated with secondary antibodies for 1 hr at room temperature. Finally, nuclei were stained with DAPI (4’,6-**d**i**a**midino-2-**p**henyl**i**ndole) for 15 min at room temperature. Post staining, cells were visualized and imaged under a fluorescence microscope (FLoid™ Cell Imaging Station).

Alkaline phosphatase assay: Live cells were treated with alkaline phosphatase staining dye following manufacturer protocols. Purple/red color is an indicator of pluripotency in the iPSC colony.

Assessment of karyotype: For qPCR based karyotyping, a commercially available kit (Stem Cell Technologies) was used per the manufacturer’s instructions to determine the status of eight commonly observed karyotype anomalies in reprogrammed iPSCs.

Mycoplasma test: Mycoplasma analysis was performed at different stages, using either of two detection methods: a Mycoplasma Detection Kit (MycoAlert, Lonza) or PCR based assessment of mycoplasma in cultured cells. The culture medium and gDNA was used as the test sample to screen for mycoplasma contamination according to manufacturer’s protocol.

Mutation analysis: Genomic DNA was extracted and purified from MLC patient-derived iPSCs. The *MLC1* region flanking the mutation site was amplified and cloned into the pcr2.1 TOPO vector. Six colonies were picked at random and the plasmid preparation was analyzed by Sanger sequencing.

## Differentiation of iPSCs to neural stem cells

0.9 to 3 million cells in single cell suspension format were counted and seeded in each well of a pre-rinsed Aggrewell 800 plate per manufacturer protocol (Stem Cell Technologies) in 2 ml iPSC media with Y27632 ROCK (**R**h**o**-associated, **c**oiled-coil containing protein **k**inase) inhibitor (10 μM, Stem Cell Technologies). The plate was centrifuged at 100 × g, 3 min, in a swinging bucket centrifuge to settle down the cells within the microwells of each well of the Aggrewell plate in a homogenous manner. Next day and onwards, generated EBs were transitioned to neural induction media: DMEM without glutamine or sodium pyruvate, N2B supplement, B27 with Vitamin A, EGF (**E**pidermal **G**rowth **F**actor, 20 ng/ml), bFGF (20 ng/ml), LDN193189 (Reagents Direct, 100 nM), 1x L-glutamine, SB431542 (Reagents Direct, 10 uM), and pen-strep (100 units/mL). Half media changes were performed every other day until day 8. On day 8, neuralized EBs were carefully picked up with help of a wide bore pipette tip and allowed to sediment with gravity. Sedimented EBs were resuspended in fresh media and were plated on a **p**oly-**L**-**o**rnithine (PLO) and laminin coated 60 mm dish. [To coat with PLO/Laminin, the following procedure was followed: on −2 day, each dish was coated with 20 ug/ml PLO in **n**uclease **f**ree **w**ater (NFW). Kept the plate at 37 °C overnight. On −1 day, removed PLO coating solution, washed 2x with NFW, followed by coating with 10 ug/ml Laminin in DPBS based solution (for plastic dishes); 20 ug/ml laminin solution was used for glass dishes, 37 °C incubator overnight. Freeze the plates in −20 and use them for upto 6 months]. The next day onwards, media was transitioned from EB media to NSC media. NSC media: DMEM (-Glutamine; -Sodium Pyruvate), 1x L-Glutamine, EGF (20 ng/ml), bFGF (20 ng/ml), B27 (− Vitamin A), 1x PenStrep. 2–3 days post EB transfer, neural rosettes were observed, but surrounded by neural crest population. Careful separation of just the neural rosettes was done by carefully plucking out the rosette centres into the media, followed by plating the rosette containing supernatant onto a fresh PLO/Laminin dish. This was considered the P0 NSC population. 2–3 days later, NSCs were accutase treated and propagated further by passaging in a 1:2 manner.

## Directed differentiation of neural stem cells to astrocytes

15,000 NSCs per cm^2^ were seeded in a T25 flask in NSC media. The next day, media was transitioned to astrocyte differentiation media consisting of astrocyte media (ScienCell Research Laboratories; Carlsbad, CA, USA; Cat. No. 1801b), astrocyte growth supplement (1x; ScienCell Research Laboratories; Cat. No. 1852), 2% Fetal Bovine Serum (FBS; ScienCell Research Laboratories; Cat. No. 0010), 50 U/ml penicillin G, 50 mg/ml streptomycin. Half medium was replaced every 3–4 days and cells were passaged every week in a 1:4 ratio on **p**oly-**D**-**l**ysine (PDL) coated dishes. After 30 days, the media was transitioned to astrocyte maturation media. Cells were then cultured for another 2–3 months depending on the experimental endpoints [41].

## RNA isolation and sequence analysis

Sample Preparation and RNA Sequencing

Total RNA was isolated from knockout (KO) and scrambled (wild type) NSCs. NSCs were collected in biological duplicates and used to prepare cDNA libraries. Samples were sequenced on an Illumina NovaSeq 6000 platform, generating paired-end 150 base pair reads.

Quality Check and Preprocessing of RNA-Seq Data

Quality control of the raw sequencing data was performed using FastQC (RRID:SCR_014583) version 0.11.9. The raw reads were trimmed to remove adapter sequences and low-quality bases using Trimmomatic (RRID:SCR_011848) version 0.39 with default settings [42]. The trimmed reads were again analyzed with FastQC (RRID:SCR_014583) and the results were consolidated using MultiQC (RRID:SCR_014982) version 1.14 [43].

Alignment and Quantification

The cleaned reads were aligned to the human reference genome GRCh38 using HISAT2 (RRID:SCR_015530) version 2.2.1 [44] with default parameters. The resulting Sequence Alignment/Map (SAM) files were converted to Binary Alignment/Map (BAM) files, sorted and indexed using SAM tools version (RRID:SCR_002105) 0.1.15 [45]. Transcript quantification was performed using feature counts, producing raw gene counts for each sample [46].

Differential Gene Expression Analysis

Differential gene expression analysis was performed using DESeq2 (RRID:SCR_015687) version 1.36.0 in R (RRID:SCR_001905) version 4.2.2 [47, 48]. Genes with an absolute log2 fold change greater than 1 and a false discovery rate (FDR) adjusted *p*-value less than or equal to 0.05 were considered significantly differentially expressed. The Enhanced Volcano package (RRID:SCR_018931) version 1.14.0 was used to visualize the differential gene expression results in a comprehensive volcano plot (RRID:SCR_025419) [49].

Functional and Pathway Enrichment Analysis

Functional enrichment analysis was conducted on the significantly differentially expressed genes using the DAVID bioinformatics resource (RRID:SCR_001881) [50] Pathway enrichment analysis was performed using KEGG (RRID:SCR_012773) [51], Reactome (RRID:SCR_003485) [52], and WikiPathways (RRID:SCR_002134) [53] databases through DAVID. Disease enrichment analysis was performed using the DisGeNET database (RRID:SCR_006178) within DAVID. The resulting disease terms were classified into different disease categories and visualized.

Protein-Protein Interaction (PPI) Analysis and Disease Association

The STRING database (RRID:SCR_005223) was used to investigate protein-protein interactions among the seven genes associated with neurological disease categories [54]. A heatmap was created to represent the enrichment of these genes in epilepsy-related diseases as obtained from the DisGeNET database.

Drug Target Analysis and ADME Prediction

The DrugBank database (RRID:SCR_002700) was used to identify potential drugs targeting the seven selected genes [55]. The drugs were filtered to only include those classified as ‘Approved’, ‘Investigational’, or ‘Experimental’. The SwissADME platform was employed to perform absorption, distribution, metabolism, and excretion (ADME) predictions on the identified drugs [56]. Investigation of drug repurposing based on synergy, ADME, and the pathways that the seven genes are involved in, are ongoing.

## Screening of *MLC1* disease-causing mutation

Genomic DNA was isolated from patient PBMCs and the patient derived iPSCs. A 271 bp amplicon of *MLC1* was amplified by PCR using the above genomic DNAs with two primer sets and amplicons were sequenced using Sanger sequencing (Primer list in Table S1).

## Results

## MLC patient-derived PBMCs were successfully reprogrammed to iPSCs

**Fig. 1:**
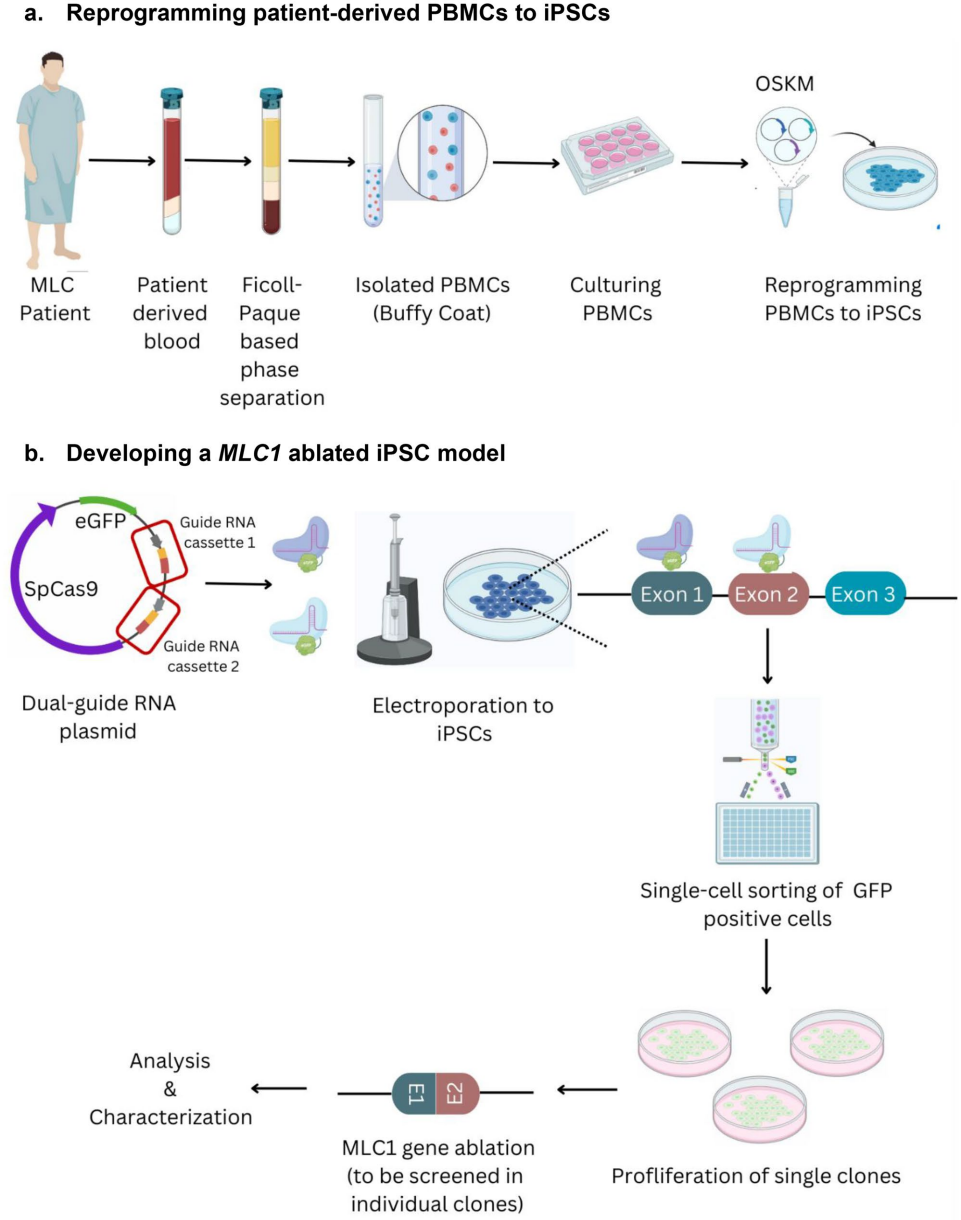
Schematic representation of methodology utilized for MLC iPSC model generation **a**. Patient-derived PBMCs were reprogrammed to iPSCs and validated for pluripotency. **b**. A *MLC1* ablated iPSC line was created via CRISPR-based editing of unaffected iPSCs and clonal isolation and screening

**Fig. 2:**
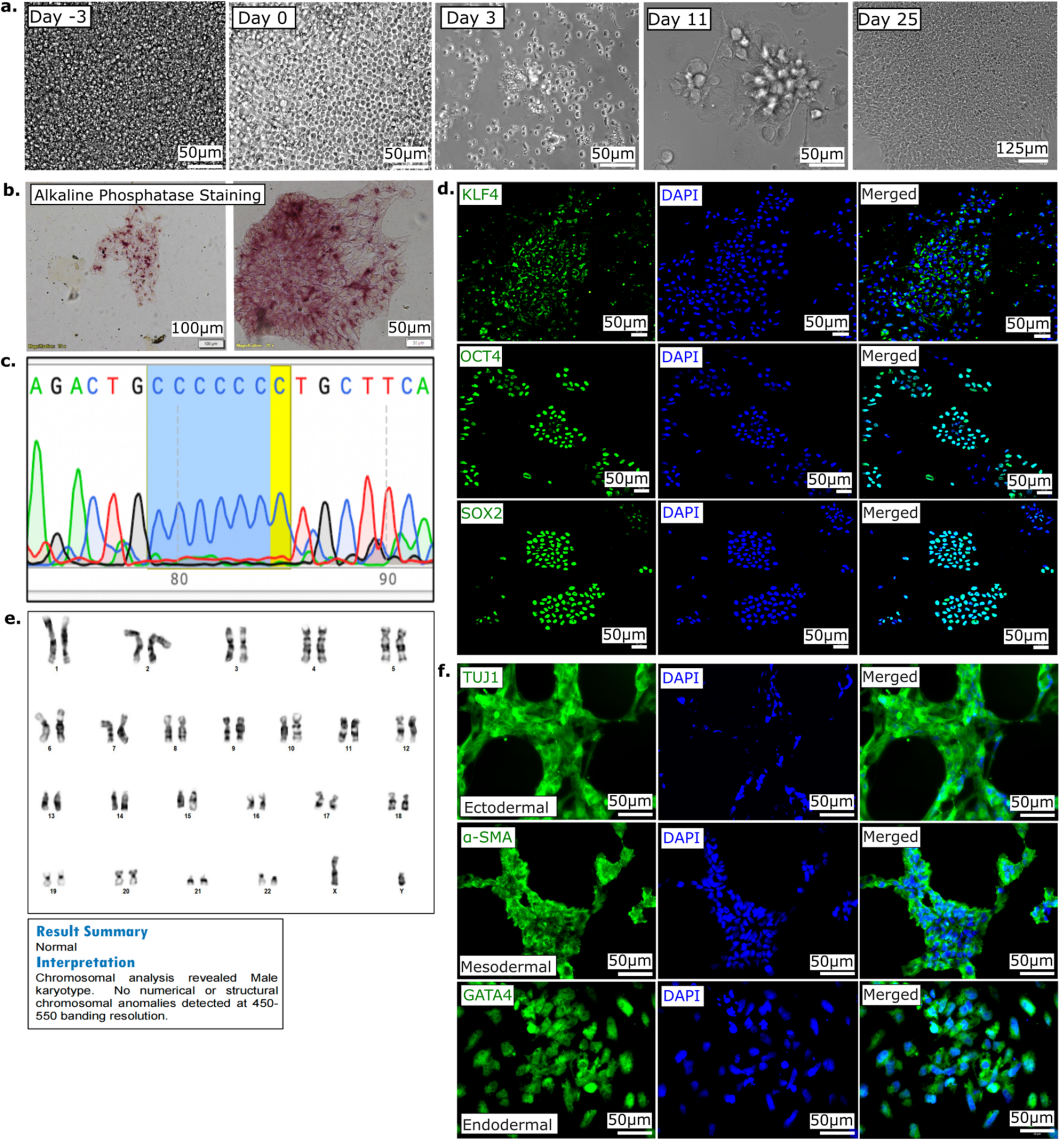
Reprogramming MLC patient PBMCs to iPSCs. **a**. sequential progression of patient-derived blood cells reprogrammed to iPSCs. **b**. derived-iPSCs were alkaline phosphatase positive. **c**. Sanger sequencing confirmation of retention of *MLC1* variant (132dupC) in iPSCs. **d**. Immunolabeling for proteins characteristic of pluripotent & proliferating iPSCs. **e**. G-banding of patient-derived iPSCs for assessment of karyotypic normalcy. **f**. immunolabeling for assessment of iPSC trilineage differentiation efficiency

To generate an MLC disease model with a closer link to human subjects, we focused on a *MLC1* mutation that produces a severe clinical phenotype. The 132dupC mutation in the Indian subcontinent affects members of the Indian Agrawal community, resulting in a mildly progressive form of MLC [16, 18]. We derived PBMCs from the MLC patient and successfully reprogrammed them to an iPSC state (Fig. 1a). PBMCs were reprogrammed using titered Sendai viral particles bearing the Yamanaka reprogramming factors. After 25 days, morphologically pluripotent cells emerged with increased volume and surface areas of some colonies, indicating successful reprogramming (Fig. 2a). Individual iPSC clones were subsequently picked, plated, propagated and cultured separately. Subsequently, a series of characterization experiments were performed to confirm that the resulting cells had indeed adopted an iPSC identity. Colonies exhibited robust alkaline phosphatase activity (Fig. 2b). To verify the patient-derived iPSCs had the desired *MLC1* genotype, we utilized Sanger sequencing to confirm the presence of the specific *MLC1* variation in the cells (Fig. 2c; Supplementary Figure S2). We also examined the expression of a suite of pluripotency markers. These markers span a range of proteins integral to maintaining the self-renewal and an undifferentiated stem cell state (Fig. 2d). We subsequently sought to ascertain the global genomic stability of our iPSCs. This assessment is critical, as the reprogramming procedure, while enabling somatic cells to revert to a pluripotent state, can also potentially induce chromosomal abnormalities [57]. We first utilized a qPCR based karyotype analysis of the iPSC clones to demonstrate a lack of karyotypic change (Supplementary Figure S3). We also employed high-resolution G (**G**eimsa)-banding to allow a comprehensive, chromosome-by-chromosome analysis of the entire karyotype. We ascertained that our iPSCs were devoid of major chromosomal anomalies, thereby confirming suitability for further research applications (Fig. 2e). Our subsequent objective was to evaluate the potential of our patient-derived iPSCs to differentiate into all three primary germ layers representing the earliest differentiation events of embryonic development and serve as the precursors to all the tissues within an organism. An assessment of tri-lineage differentiation in our patient-derived iPSC line is a key criterion for validating pluripotency. Following a standardized differentiation protocol, we verified successful lineage commitment by staining the cells for a set of lineage-specific markers representing the ectodermal, mesodermal, and endodermal lineages. The presence of these markers post-differentiation demonstrated their capacity to differentiate into all three germ layers, thereby further substantiating their pluripotent state (Fig. 2f).

## Creation of a genetically ablated *MLC1* line

**Fig. 3:**
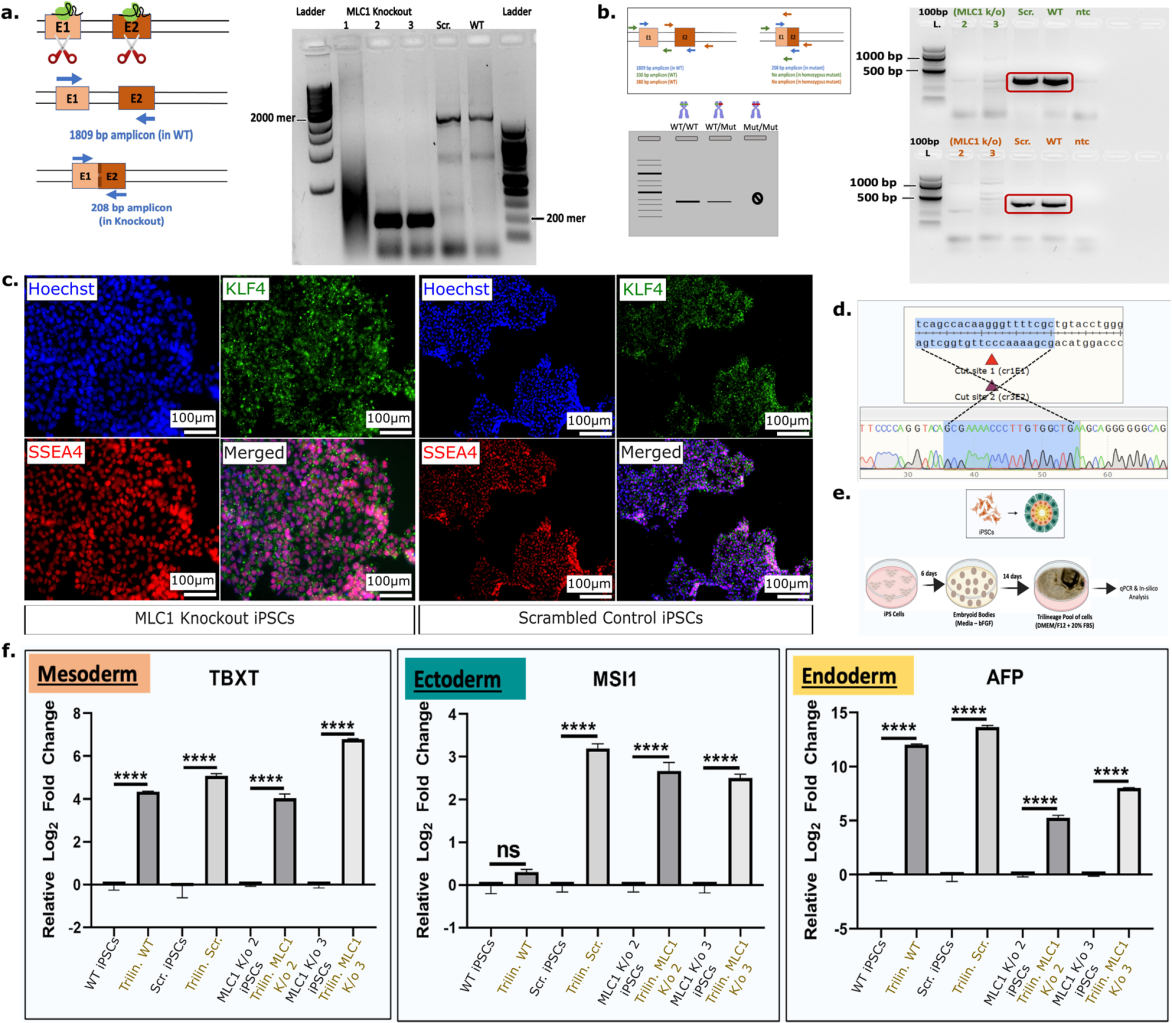
Generation of *MLC1* knockout iPSCs. **a**. The two ribonucleoprotein complexes from the dual guide plasmid target exon 1 and exon 2 of *MLC1*, resulting in a truncated 200 bp amplicon compared to a ~ 2000 bp amplicon in wild-type control. **b**. PCR based genotyping of clones *MLC1* knockout 2 & 3 demonstrate biallelic deletion of *MLC1*. **c**. *MLC1* ablated iPSCs express markers of pluripotency. **d**. Representative Sanger sequencing assessment of gRNA targeted *MLC1* in clone *MLC1* knockout 2. **e**. Schematic of trilineage differentiation of *MLC1* knockout, WT, & scrambled control iPSCs. **f**. *MLC1* knockout iPSCs could be differentiated to all three germ layers (as good as the WT & scrambled control iPSCs) as seen by qPCR assay (performed two-tailed, Student’s t-test; **** *p*-value < 0.05)

Considering the broad clinical phenotypes of MLC, iPSCs derived solely from patients, though invaluable, may not encapsulate the entirety of the disease pathology at a cellular level. To analyze MLC impacts beyond a specific gene variant, we utilized a CRISPR-Cas9 system in a wild-type iPSC line to create a *MLC1* knockout exhibiting completely inactivated *MLC1* and providing a complementary model system for studying the effects of complete loss of MLC1 function. We cloned a **g**uide **RNA** (gRNA) sequence targeting *MLC1* into a mammalian expression plasmid. This plasmid construct contained a cassette for the expression of two gRNAs, a *Streptococcus pyogenes* derived **Cas9** (SpCas9) gene cassette, and a green fluorescent reporter cassette. The two gRNAs were designed to target distinct regions in *MLC1* to enhance the introduction of multiple double-strand breaks (Fig. 1b). We transfected HEK293T (**H**uman **E**mbryonic **K**idney cells generated at **293**^rd^ experiment after transfection with Simian Virus 40 large **T** antigen) cells with different combinations of gRNAs in the dual gRNA plasmid to test the genome editing efficiency of the plasmid construct in the pool population (Supplementary Figure S4). Following electroporation in iPSCs, we carried out single-cell sorting to isolate individual cells for propagation into distinct clones. Upon sufficient proliferation of these clones, we then screened clones using a genotyping-based PCR to assess *MLC1* deletion in the iPSCs. We successfully identified two clones (signified as *MLC1* knockout 2 and *MLC1* knockout 3) that demonstrated successful Cas9-mediated *MLC1* deletion (Fig. 3a). Upon identifying the two clones that displayed the desired genotype, we aimed to further investigate whether *MLC1* deletion occurred in a biallelic manner. We utilized an alternative genotyping strategy in which we designed primer pairs that flanked exons 1 and 2 of *MLC1*. In case of genetic ablation in monoallelic manner, we should get bands at the size of 330 bp and 380 bp with different primer sets, whereas if it were to have happened in a biallelic manner, there should be no band at all (as the genetic ablation would have resulted in the deletion of the *MLC1* region, resulting in an inability of one of the primer set to bind and give rise to an amplicon). As per our observations, both iPSC clones did not show any amplification, thereby suggesting potentially successful biallelic knockout of *MLC1* in these iPSC clones (Fig. 3b). We subsequently performed Sanger sequencing which corroborated our PCR screening, conclusively confirming that a biallelic knockout of *MLC1* had occurred in our iPSC clones (Fig. 3d). Following confirmation of *MLC1* knockout in our iPSC clones, we proceeded to carry out characterization for pluripotency in *MLC1* deleted iPSCs and isogenic scrambled controls. To evaluate pluripotency, we utilized an immunofluorescence assay for the expression of pluripotency markers OCT4 and SSEA4. *MLC1* knockout clone retained pluripotent protein expression, suggesting *MLC1* loss does not impact pluripotency (Fig. 3c). We next examined the trilineage differentiation capacity of the *MLC1* knockout iPSC line. To achieve this, we induced undirected differentiation of the iPSCs by permitting cellular aggregation and spontaneous differentiation into embryoid bodies over 21 days (Fig. 3e). qPCR analyses validated the trilineage differentiation capacity of *MLC1* deleted iPSCs (Fig. 3f).

## Patient-derived and *MLC1* deleted iPSCs successfully differentiate to neural stem cells

**Fig. 4:**
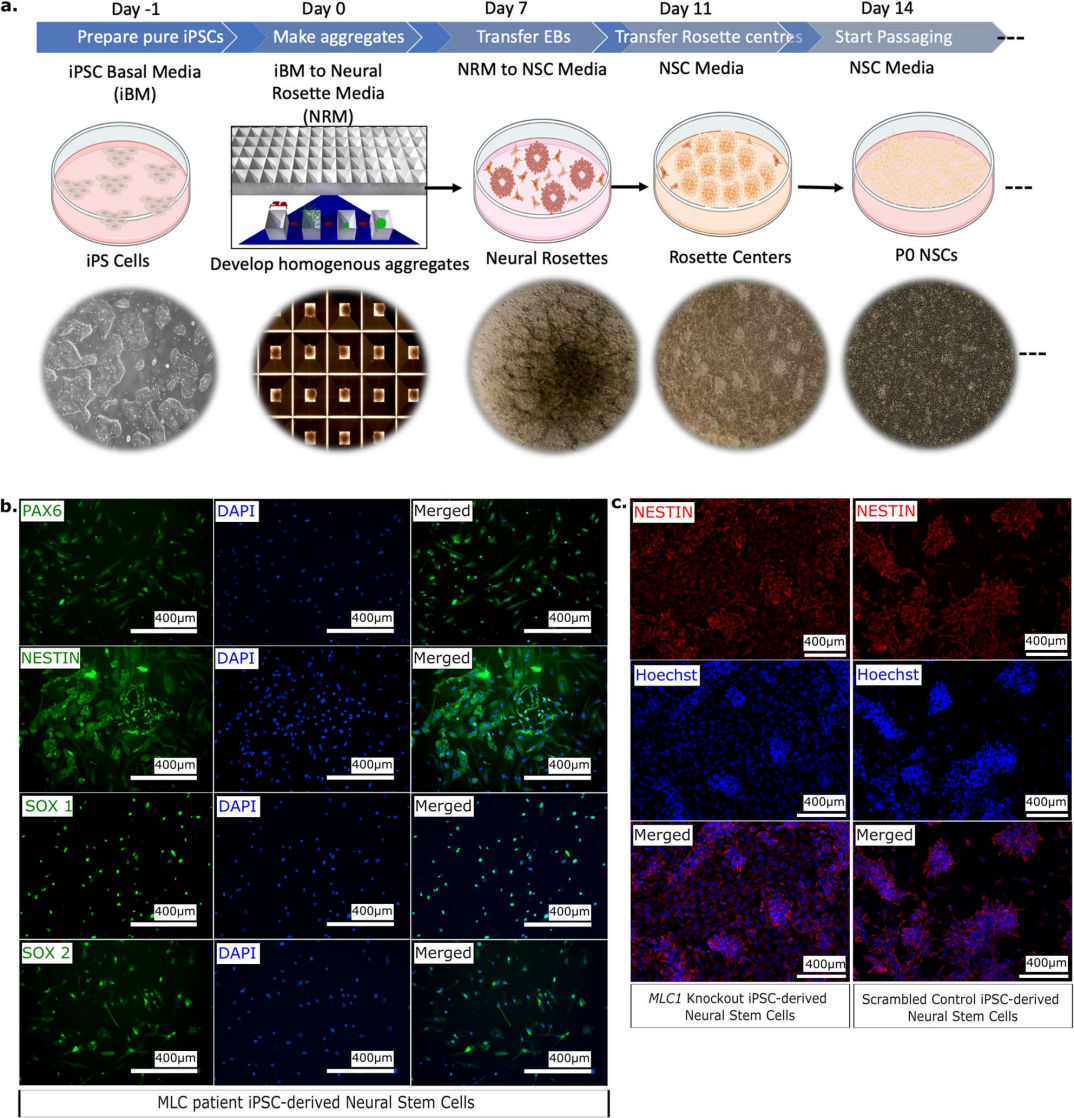
Generation of neural stem cells from MLC patient-derived iPSCs and *MLC1* knockout iPSCs. **a**. Schematic representation and microscopic (bright-field) depiction of protocol of NSC generation. **b**. Immunostaining of neural stem cells derived from patient-derived iPSCs. **c**. Immunostaining of neural stem cells differentiated from *MLC1* and scrambled control iPSCs

To assess the impact of *MLC1* mutation on neurodevelopment, both patient-derived and *MLC1* knockout iPSCs were differentiated into neural stem cells (NSCs), a population of self-renewing, multipotent progenitors with the potential to differentiate into neurons, astrocytes, and oligodendrocytes. We speculated that studying *MLC1* knockout NSCs might give us glimpses of potential pathways that eventually lead to disease manifestation in glia. We utilized a detailed differentiation protocol that guides the pluripotent stem cells through sequential stages of differentiation, ultimately leading to the formation of NSCs (Fig. 4a).

The differentiated NSCs that were derived from the patient derived iPSCs (Fig. 4b), *MLC1* knockout, and scrambled control iPSCs (Fig. 4c) were immunolabeled with NSC specific antibodies (PAX6 (**pa**ired bo**x** protein **6**), Nestin (**ne**uroepithelial **st**em cell prote**in**), SOX1 (**S**RY-b**ox** transcription factor **1**; (SRY (**S**ex-determining **r**egion **Y**)), SOX2 (**S**RY-b**ox** transcription factor **2**)). The presence of the respective fluorescence signals in these cell populations provided us with compelling evidence of successful NSC generation.

## Transcriptomic profiling of MLC patient-derived and *MLC1* neural stem cells reveals disease-relevant differentially expressed genes

**Fig. 5:**
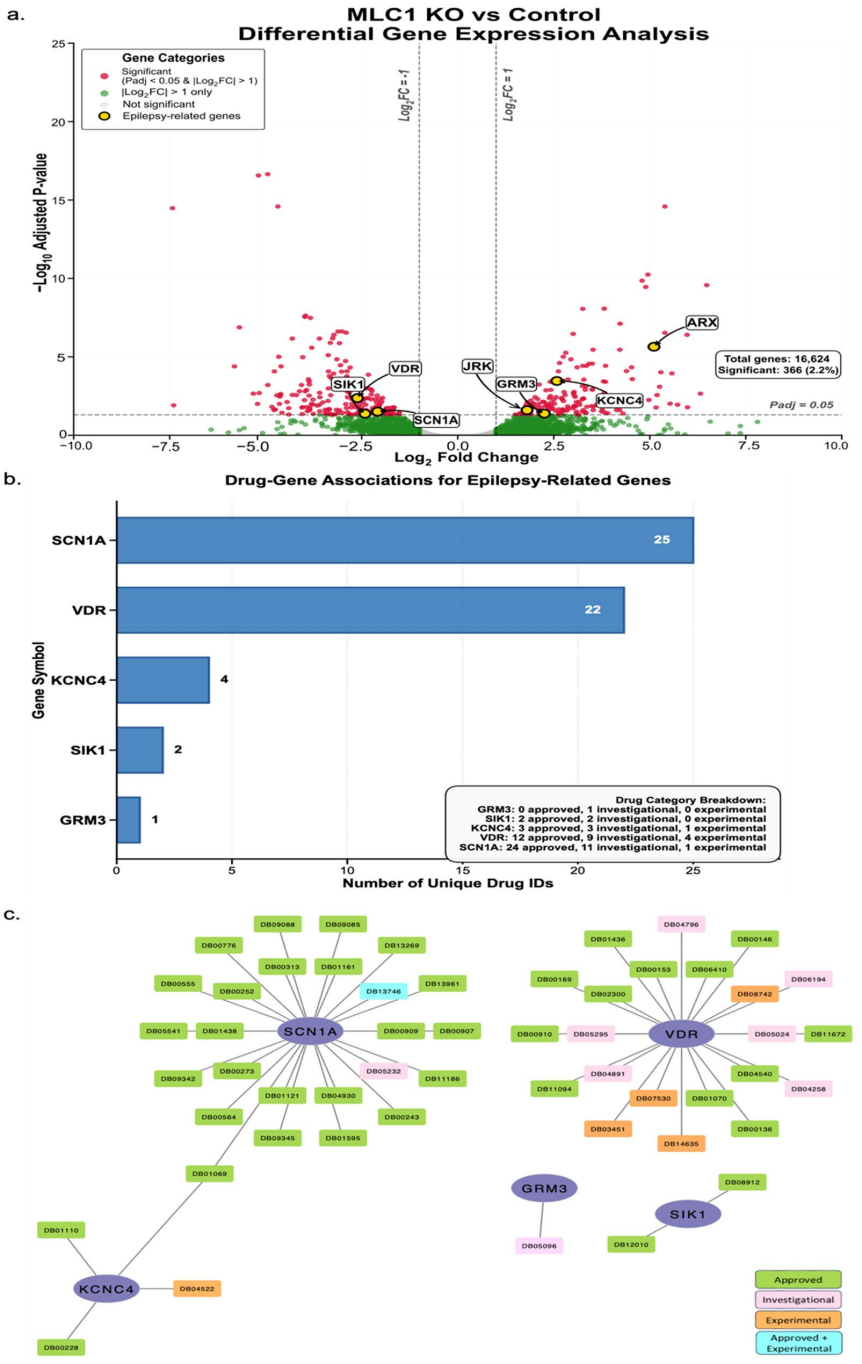
Transcriptomic profiling of neural stem cells derived from *MLC1* deleted iPSCs. **a**. Volcano plot depicting differentially expressed genes in *MLC1* knockout NSCs compared to isogenic controls. Epilepsy related genes are highlighted. **b**. Histogram depicting numerical estimate of a comprehensive list of drugs associated with epilepsy related genes. **c**. Identification of drugs targeting epilepsy-related genes that might be investigated and repurposed for MLC therapy. Green colored drugs are the approved category of drugs, pink is for investigational drugs, blue is for approved+ experimental, orange is for experimental drug category, purple is for genes

MLC1 expression is predominantly associated with later stages of neural differentiation, specifically when NSCs undergo lineage specification towards astrocytes [58, 3]. However, MLC1 might play an unidentified role in earlier stages of neurodevelopment before astrocyte lineage commitment. NSC transcriptome profiling might offer an opportunity to unveil early pathological markers or identify signaling pathways associated with MLC pathogenesis. Our results revealed a relatively small number of differentially expressed genes (366 total). The differentially expressed genes were primarily associated with neurological disorders including epilepsy (Fig. 5a

; Supplementary Table S4). The overall modest number of differentially expressed genes may hint at subtle but crucial alterations in cellular pathways that contribute to the pathogenesis of MLC, including expression of the epilepsy-associated genes identified by RNA sequencing (Fig. 5a) and a qPCR based validation of these findings in few of the epilepsy-related genes, amongst the top 10 upregulated & top 10 downregulated ones from our RNA sequencing results (Supplementary Figure S6a, b). In order to gain more insightful interpretations from our dataset, we classified the differentially expressed genes according to diseases in which they have been implicated (Supplementary Figure S5). Surprisingly, the neurological diseases and their corresponding genes identified within our transcriptomic data were all associated with epilepsy, a condition commonly observed in MLC patients. This unexpected link at the NSC stage underscores the potential link between MLC and epileptogenic pathways, suggesting that it might be plausible that MLC’s clinical presentation could be influenced by disturbances in the neuronal circuits associated with epilepsy. Concurrently, we conducted an exhaustive review of experimental, investigational, and approved therapeutics to identify potential drug candidates that could be repurposed for MLC treatment based upon identified epilepsy-associated pathways (Fig. 5b,**c**). Beyond identifying the potential signaling pathways of interest to MLC, these data identified within NSCs also suggest that MLC pathogenesis may involve both developmental defects and astrocyte dysfunction. However, an additional profiling of astroglial lineage transcriptomics is needed to establish a unified model of MLC pathogenesis, and the present findings lay the groundwork for the experimental validation and drug-screening efforts that must follow.

*MLC1* **knockout iPSCs successfully differentiate to disease-relevant astrocytes:**

**Fig. 6:**
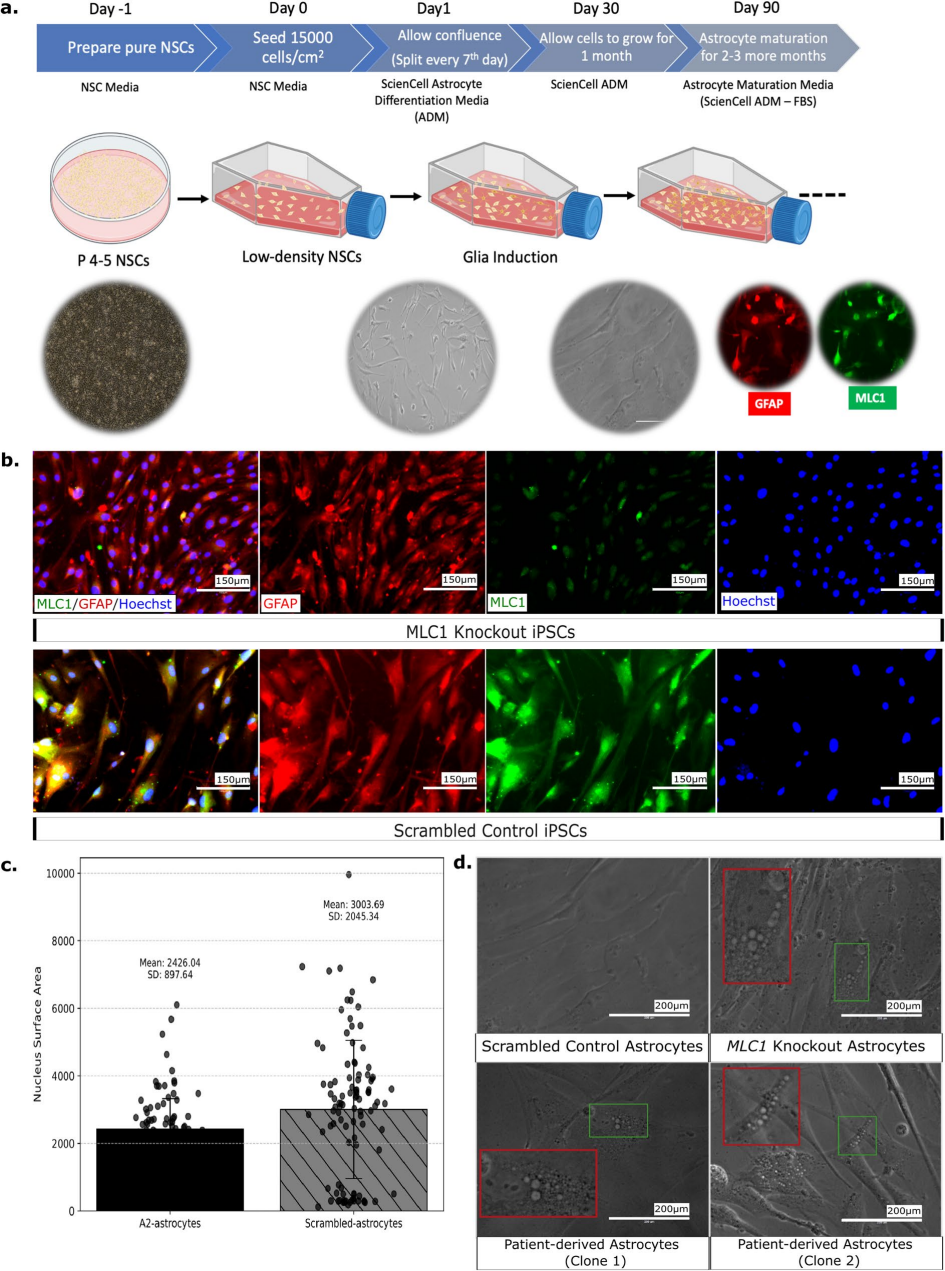
*MLC1* differentiated astrocytes exhibit a vacuolation phenotype characteristic of MLC. **a**. Schematic representation and microscopic (bright-field) depiction of the protocol used for astrocyte generation. **b**. MLC1 expression is ablated in *MLC1* knockout astrocytes but highly expressed in controls. 2015” ia_version=“0”>**c**. The cellular nuclei of *MLC1* knockout astrocytes have a smaller surface area compared to scrambled control (mann-whitney U test; *p*-value = 0.0017; *n* = 101). **d**. *MLC1* knockout and patient-derived astrocyte cell lines displayed vacuoles (red inset shows magnification of the area enclosed by green inset), as previously observed in *MLC1* rat astrocytes [10]

MLC1 has a specific and high expression in astrocytes [3, 59] (Figure S1). To model the impact of MLC1 on astrocyte biology, we followed a standardized protocol to differentiate NSCs derived from control, MLC patient-derived, and *MLC1* knockout iPSCs to an astrocyte lineage [60]. This optimization included a series of procedures and conditions tailored to foster the appropriate differentiation of these distinct NSC lines into astrocytes (Fig. 6a). In the process of astrocyte differentiation, we consistently monitored for morphological changes as a preliminary measure of progression and the success of the differentiation process. By day 30, we observed that the cells had begun to express characteristic astrocyte markers, including glial fibrillary acidic protein (GFAP), indicating their successful differentiation into astrocytes. In addition, the MLC1 protein also began expressing in the cells. However, as predicted, the MLC signal was seemingly diminished in the *MLC1* gene knockout cell line (Fig. 6b).

**Fig. 7:**
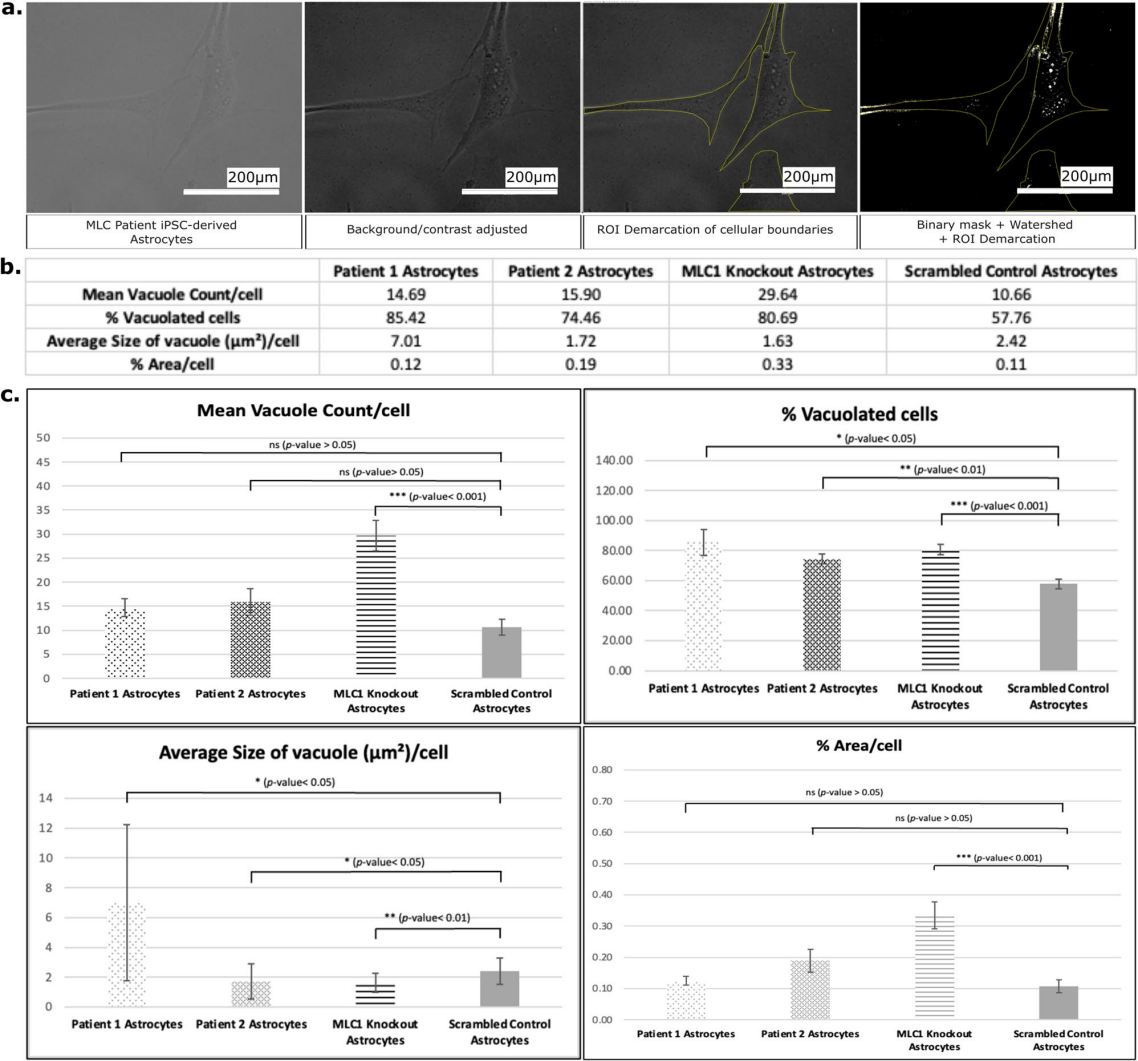
Analyses of vacuolation in MLC patient and MLC1 knockout iPSC-derived astrocytes **a**. Representative images showing the demarcated cellular structures. **b**. Mean count of the different parameters (including vacuoles per cell, the percentage of vacuolated cells, the average size of vacuoles, and the percent vacuolated area per cell) is represented. Analysis was done on 4 different image panels for patient 1 astrocytes, 11 panels for patient 2 astrocytes, 8 panels for MLC1 knockout astrocytes, and 12 for scrambled control astrocytes. **c**. Graphical representation of the different parameters. Error bars represent standard error of mean. Pairwise, two-tailed, two sample unequal variance (heteroschedastic) t-test *p*-values are shown

During the course of differentiation, we noted the emergence of vacuolation across the astrocyte cell body of both MLC patient-derived and *MLC1* knockout astrocytes. This phenotypic characteristic, a hallmark of MLC pathology, was distinctly absent in control astrocytes (Fig. 6d). The appearance of these vacuoles is noteworthy, as it mirrors the phenotype observed in patient brain tissue. Upon image analyses, we found a significant increase in the average number of vacuoles per cell, the percentage of vacuolated cells, the average size of vacuoles, and the percent of vacuolated area per cell in MLC1 knockout iPSC derived astrocytes compared to control iPSC derived astrocytes (Fig. 7).

Cellular vacuoles can be acidic in nature, such as lysosomes, while others can be neutral or basic. To rule out that the morphological MLC vacuoles are lysosomes, we examined if the vacuoles that we observe in MLC astrocytes are acidic. We used LysoTrackerRED, a specific and widely-used fluorescent probe designed for the purpose of labelling and tracking acidic organelles within live cells. Our data revealed that there is little to no colocalization between the LysoTrackerRED dye and the vacuoles within our cells (Figure S8a, b). This evidence refutes that a major lot of the observed vacuoles are acidic in nature, thereby ruling out the possibility of them being lysosomal or other acidic vesicles. The recapitulation of this disease-specific vacuolar phenotype strongly affirms that our MLC iPSCs represent a robust model for MLC. Parallelly, we observed that the cellular and nuclear size of the *MLC1* knockout iPSCs was shrunken in size as compared to the scrambled control iPSCs (Figure S7, Fig. 6c).

## Discussion

MLC is a disease that manifests due to mutations in *MLC1* or *GlialCAM* in an autosomal recessive or an autosomal dominant manner. To better delineate MLC pathogenesis, we developed novel iPSC models of MLC due to either a specific *MLC1* mutation (132dupC) found in the Indian Agrawal community or using CRISPR/Cas9 system to create *MLC1* knockout iPSCs. Upon validation of the iPSC lines via multiple parameters to affirm their iPSC status, we also differentiated these cells into disease-relevant neural lineages. We performed transcriptomic profiling of *MLC1* mutant and control neural derivatives, uncovering > 350 differentially expressed genes associated with neurological disease, including epilepsy. During the course of astrocyte differentiation, we also found vacuolation within the cell bodies of MLC patient-derived and *MLC1* knockout astrocytes, a feature absent from isogenic controls. These data demonstrate that derived MLC iPSCs represent a valid model system capable of recapitulating key hallmarks of MLC disease.

To study MLC pathophysiology, various animal models have been developed. *MLC1* and *GlialCAM* knockouts, including several knockout (KO) and knock-in (KI) models, have been created in mice [11, 61–65], as well as zebrafish [29, 63]. These models share similarities with human MLC patients, such as increased brain water content and intramyelinic vacuoles, detectable by MRI and histological methods [1]. However, when compared with humans, many major differences exist, including the timing of MRI-detectable defects that occur during the initial stages of life in human patients while reducing and gaining stability at later stages [66, 67]. Also, there have been observable regional variations in vacuolization. For human subjects, the subcortical white matter region sustains brain edema. Within MLC mouse models, the pathological defects are mainly observed within the cerebellum [67, 68]. There are also substantial cellular and functional disparities that exist between human and rodent astrocytes [69]. The human brain exhibits a higher astrocyte-to-neuron ratio compared to rodents, underscoring the importance of deriving astrocytes from a human source using an effective protocol to study human-specific disease states. To circumvent these issues and to complement animal model based studies, there have been studies conducted on lymphoblast cell lines and human monocytes or their derived macrophages from wild-type and MLC patients [26, 70]. In this scenario, the patient-derived and genetically ablated iPSC based models of MLC disease would add further to the repertoire of MLC disease research and understanding of the disease pathology. The emergence of MLC-related gene expression in early stage neural stem cells and the prevalence of disease-like hallmarks (vacuolation) in differentiated astrocytes provides a glimpse of MLC disease progression during development.

Several leukodystrophies have also been modeled using patient-derived or genetically engineered iPSCs. *GFAP*-mutant iPSC-derived astrocytes recapitulated the pathological features of Alexander disease: Rosenthal-fiber pathology and impaired oligodendrocyte maturation, while CRISPR correction rescued these defects [71, 72]. Similarly, iPSC models of Vanishing White Matter disease showed that *EIF2B* mutations produce structurally abnormal, stress-sensitive astrocytes [73] that disrupt **O**ligodendrocyte **P**recursor **C**ell (OPC) development [74]. In Pelizaeus–Merzbacher disease, *PLP1* (**p**roteo**l**ipid **p**rotein 1) mutant iPSC-derived oligodendrocytes revealed ER stress, ferroptosis, and differentiation failure [75], while gene correction and iron chelation restore cell viability and myelination [76]. These studies highlight how human glial models unveil cell-autonomous dysfunction that animal models do not. In conclusion, iPSC-based leukodystrophy disease models offer a species-appropriate glia-centered model system that compensates for interspecies differences with established animal-based model systems and compliments the end goal to understand disease mechanisms and development of therapeutics.

Based upon the identification of drug candidates associated with differentially expressed genes from our dataset, it may be possible to repurpose available investigational or approved drugs targeting epilepsy-related genes for potential therapeutics for MLC. Similar drug screening studies in iPSC-derived neural models have been performed for a variety of rare and common neurological diseases. Lee et al. [77] modeled the pathogenesis and treatment of familial dysautonomia with derived neural crest precursors from patient-specific iPSCs. [78] modeled neural development and treatment strategies for Rett syndrome as a model of autism spectrum disorder, using patient iPSCs-derived neurons. [79] developed an anti-Aβ (**A**myloid beta(**β**)) drug screening platform using iPSC-derived neurons to treat Alzheimer’s disease. [80] used patient-derived Niemann–Pick disease type C neural stem cells as a model system for evaluation of drug efficacy and study of the disease pathogenesis. [81] and [82] developed a robust high-content screening assay to identify compounds that reduce tau levels and toxic Aβ levels in iPSC derived neurons, as a potential therapy for Alzheimer’s disease. Similar to these published studies, we propose that our MLC iPSC models and differentiated neural derivatives represent an ideal cellular platform for screening drugs and small molecules which inhibit hallmarks of MLC disease for eventual translation to MLC patients.

Moving forward, future analysis of these candidate genes, their signaling pathways and its association with MLC will be critical. Since the neural cells in MLC display a dysregulation in astrocyte functionality, a thorough study of MLC astrocyte function could be one of the proposed areas to research. Several groups have worked in this area but in different model systems that can be repurposed for our *in-vitro* disease model too. One such functionality test is monitoring shifts in cellular volume due to water influx/efflux. A dysregulation in certain ion channels has been associated with conditions such as cerebral edema and brain inflammation [83]. An *in-vivo* chemical labelling of mouse brain with an astrocyte specific red fluorescent dye called **s**ulfo**r**hodamine **B** (SRB) has been performed where fluorescence changes in astrocytes reflect volume oscillation because of transmembrane water flux changes [84, 85]. This study helped decipher the role of AQP4 (**Aq**ua**p**orin **4**) protein in maintaining water efflux properties to maintain brain homeostasis [86]. Additionally, mice found deficient in connexin gap junction proteins disrupt astrocyte connectivity resulting in a spike in hippocampal synaptic transmission, impaired astroglial glutamate and potassium clearance, and regulation of extracellular space volume which eventually led to a decrease in their long term synaptic plasticity [87]. One study used molecular, physiological, and modelling approaches to understand the mechanistics of astroglial network regulation of hippocampal bursting patterns and identified the protein KCNQ (potassium(**K**^+^) voltage-gated **c**hannel subfamily **Q**) as the downstream or the molecular target of astroglial gap junctions, with a therapeutic potential for the disease [88]. It is also noted that another major function of astrocytes in the brain is to encapsulate the brain vasculature via **p**eri**v**ascular **a**strocyte **e**nd**f**eet (PV-AEF) to give rise to the **n**euro**v**asculature **u**nit (NVU) [89, 90]. It has also been noted that ablation of *MLC1* in perivascular astrocytes, which help maintain the integrity of the blood brain barrier [91], causes barrier break down, as indicated by protein leakage and decreased expression of **c**ell **a**dhesion **m**olecule**s** (CAMs) in **v**ascular **e**ndothelial **c**ell**s** (VECs) [92]. While most of these studies were conducted in mouse models, similar studies performed in our *in-vitro* human model system of MLC could help develop a deeper understanding of disease-associated mechanisms.

## Conclusions

In conclusion, we have developed well characterized MLC patient-derived and *MLC1* mutant iPSC models of Megalencephalic Leukoencephalopathy with subcortical cysts. These cellular models can serve as a platform for a variety of screening assays mediated by chemical and genetic interventions, with a strong potential of discovery of a therapeutic solution for MLC and to help better understand the pathogenesis of this disease. In particular, a detailed analysis of the hallmark MLC vacuolation and an in-depth characterization of MLC iPSC-derived astrocytes can serve as a phenotypic assay for drug screening platforms or gene augmentation assays to develop or repurpose drugs to improve the lives of MLC patients.

## Electronic supplementary material

Below is the link to the electronic supplementary material.

Supplementary material 1

Supplementary material 2

## Data Availability

The authors confirm that the data & materials supporting the findings of this study are available within the article [and/or] its supplementary materials. Further inquiries can be directed to the corresponding author.

## Abbreviations

132dupC: *MLC1* gene mutation with a cytosine duplication, found in the Agrawal cohort with MLC.
22qtel: Telomeric end of long arm (q) of chromosome 22.
AFP: Alfafeto protein
Alpha-SMA: Smooth muscle alpha-actin
AQP4: Aquaporin 4
ARX: Aristaless-related homeobox
bFGF: Basic fibroblast growth factor
BSA: Bovine Serum Albumin
ClC2: Chloride channel 2 gene
CNS: Central Nervous System
CRISPR: Clustered Regularly Interspaced Short Palindromic Repeats
DMEM: Dulbecco’s Modified Eagle Medium
DNA: Deoxyribonucleic Acid
DSB: Double Strand Breaks
EGF: Epidermal growth factor
ESC: Embryonic Stem Cells
ER: Endoplasmic Reticulum
ERAD: Endoplasmic Reticulum Associated Degradation
FACS: Fluorescence Activated Cell Sorting
FLT3: Fms (Feline McDonough Sarcoma)-like tyrosine kinase 3
FBS: Fetal Bovine Serum
G-banding: Giemsa banding
GFAP: Glial Fibrillary Acidic Protein
GFP: Green Fluorescent Protein
GlialCAM: Hepatic and Glial Cell adhesion molecule
gq-PCR: Genomic qPCR
GRM3: Glutamate Metabotropic Receptor 3
gRNA: guide RNA
HDR: Homology Directed Repair
HEK293: Human embryonic Kidney
HEK293T: Human embryonic Kidney 293T
HepaCAM: Hepatic and Glial Cell adhesion molecule
IL-3: Interleukin 3
IL-6: Interleukin 6
indel: Insertion/deletion
iPSC: Induced Pluripotent Stem Cells
KCNC4: Potassium Voltage-Gated Channel Subfamily C Member 4
KCNQ: potassium(K^+^) voltage-gated channel subfamily Q
KEGG: Kyoto encyclopedia of genes and genomes
Kir 4.1: Inwardly rectifying potassium
Klf4: Krüppel-like factor 4
KO: Knockout
MLC: Megalencephalic leukoencephalopathy with subcortical cysts
MLC1: Megalencephalic leukoencephalopathy with subcortical cysts 1
MRI: Magnetic resonance imaging
mRNA: Messenger RNA
MSI1: Musashi RNA binding protein 1
MwoI: MwoI gene from *methanobacterium wolfeii*
Na K-ATPase: Sodium potassium adenosine triphosphate
Nestin: Neuroepithelial stem cell protein
NGS: Next generation sequencing
NPC: Neural progenitor Cells
NSC: Neural stem cells
Oct 4: Octamer-binding transcription factor 4
OPC: Oligodendrocyte precursor cell
OSKM: Yamanaka factors (Oct3/4, Sox2, Klf4,c-Myc)
PAX6: Paired box protein 6
PBMC: peripheral blood mononuclear cells
PBS: Phosphate buffer saline
PCR: Polymerase chain reaction
PDL: poly-D-Lysine
PINK1: PTEN induced putative kinase 1
PLO: poly-L-ornithine
PLP1: proteolipid protein 1
qPCR: Quantitative polymerase chain reaction
ReLeSR: Reagent for enzyme-free Lifting of embryonic Stem cell Regions
RFLP: Restriction Fragment Length Polymorphism
RN: A Ribonucleic Acid
RNP: Ribonucleoprotein
ROCK: Rho-associated, coiled-coil containing protein kinase
SCF: Stem Cell factor
SCN1A: Sodium channel protein type 1 subunit alpha
SDM: Site-Directed Mutagenesis
SFM: Serum free media
sgRNA: Single guide RNA
SIK1: Salt Inducible Kinase 1
siRNA: Small interfering RNA
SOX1: SRY-Box Transcription Factor 1; SRY (Sex-determining region Y)
SOX2: SRY-Box Transcription Factor 2; SRY (Sex-determining region Y)
SpCas9: Cas9 variant derived from the bacterial species Streptococcus pyogenes
SSEA4: Stage-specific embryonic antigen-4
TBXT: T-box transcription factor T
TRPV4: Transient receptor potential vanilloid-type 4
V-ATPase: Vacuolar-type ATPase
VDR: Vitamin D receptor
WHO: World health organisation
WT: Wild type
ZO-1: Zonula occludens-1
