# Supplementary material for: MLC1 alteration in human iPSCs give rise to disease-like cellular vacuolation phenotype in the astrocyte lineage
Source: Orphanet J Rare Dis. 2026 Mar 26;21:176. doi: 10.1186/s13023-026-04316-3 (PMC13147569; doi:10.1186/s13023-026-04316-3)
Supplement: Supplementary file 1 — Supplementary material 1 [file 13023_2026_4316_MOESM1_ESM.docx]

**SUPPLEMENTARY INFORMATION (FIGURES):**


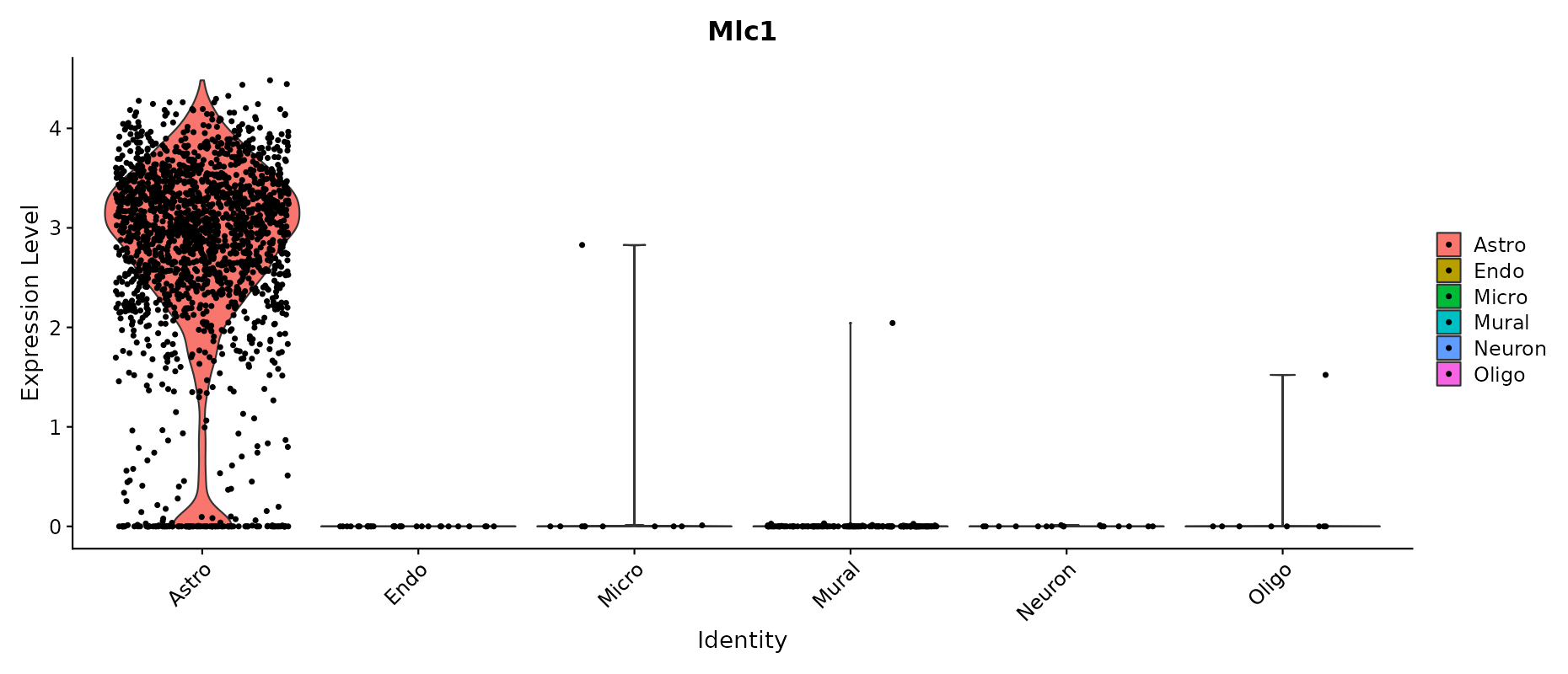

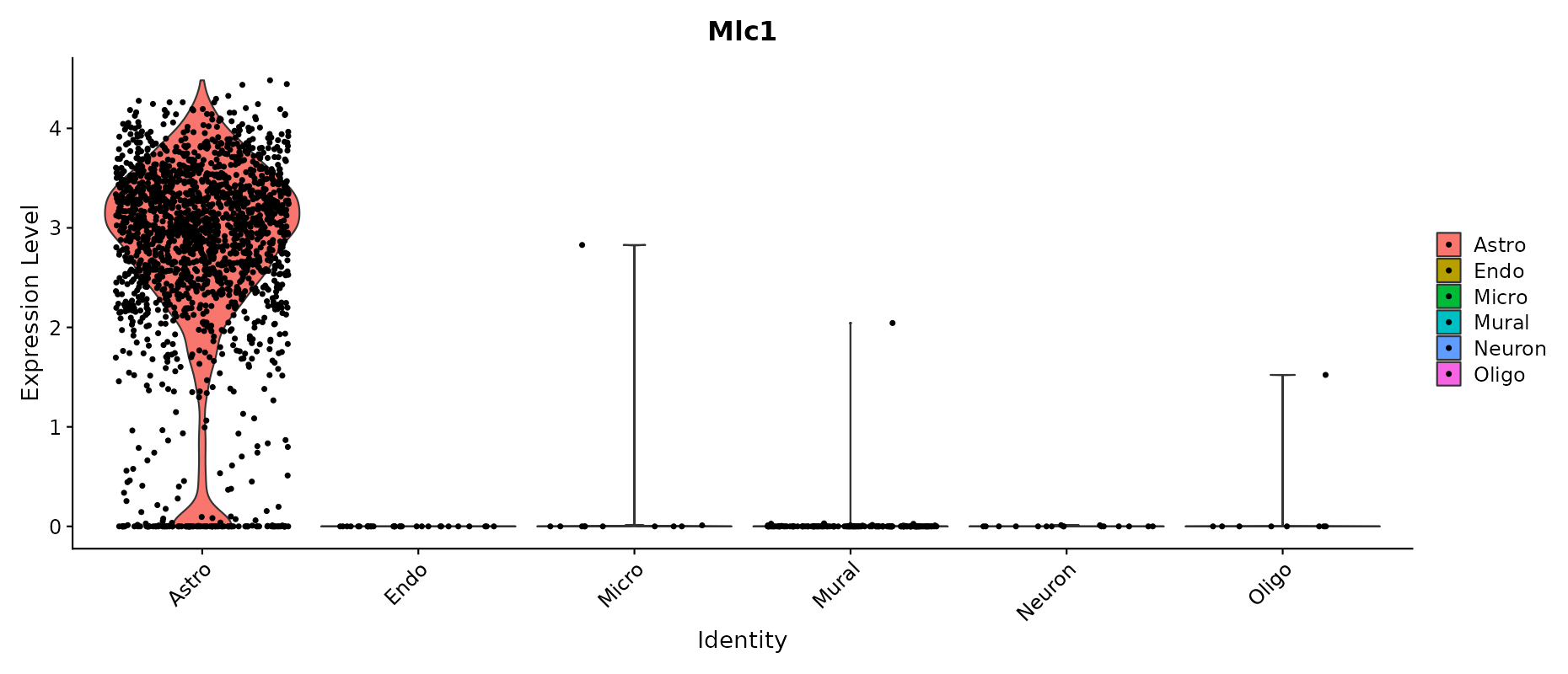


Astrocytes

Endo

Micro

Mural

Neuron

Oligo

**Figure S1. Astrocytes exhibit nearly exclusive expression of *MLC1* within human brain cells (as curated from:** [**https://holt-sc.glialab.org/sc/**](https://holt-sc.glialab.org/sc/) **)**

**Figure S1. Astrocytes exhibit nearly exclusive expression of *MLC1* within human brain cells (as curated from:** [**https://holt-sc.glialab.org/sc/**](https://holt-sc.glialab.org/sc/) **)**


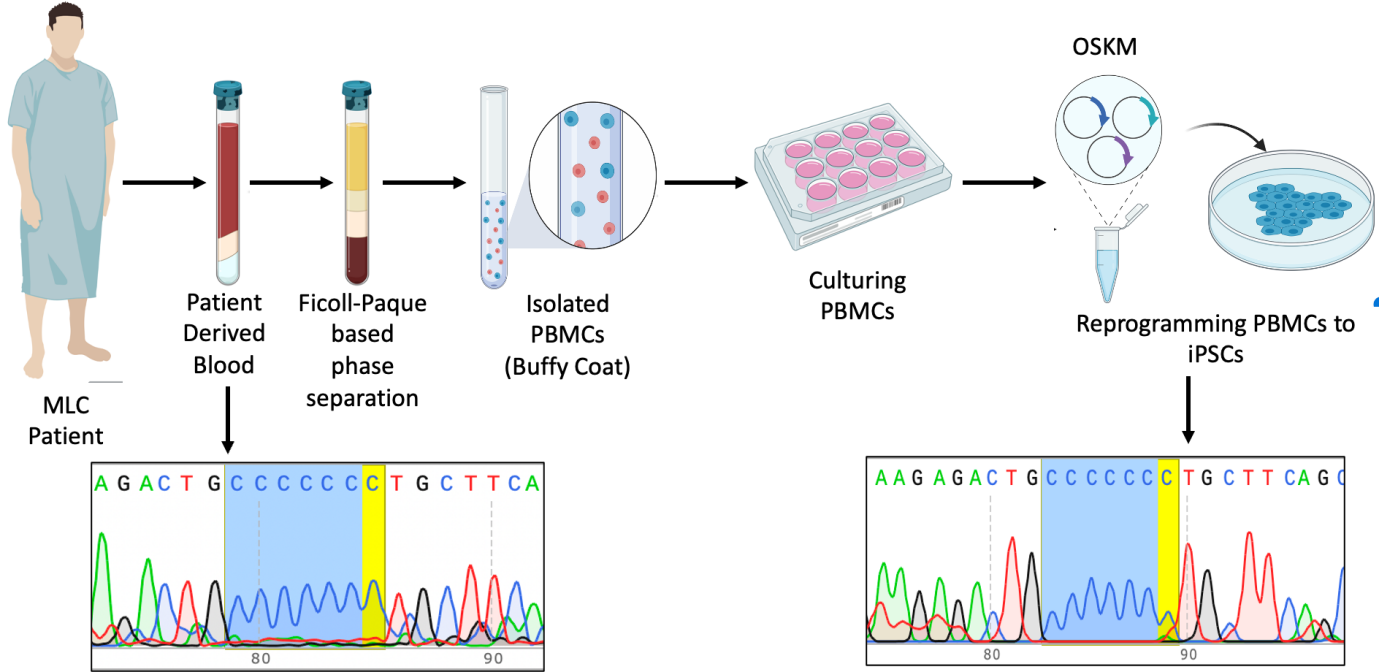


**Figure S2. Sanger sequencing was performed with *MLC1* gene amplicon from gDNA isolated from patient-derived blood as well as the cells obtained post-reprogramming.** Sequencing demonstrated that the insertion mutation (136dupC) resulting in MLC is prevalent in the patient-derived blood cells (towards the left side) as well as the reprogrammed cells (towards right side). This estimation helps confirm that the reprogramming procedure didn’t affect the integrity of gene locus of our concern, i.e. *MLC1* region which bears the patient mutation.

**
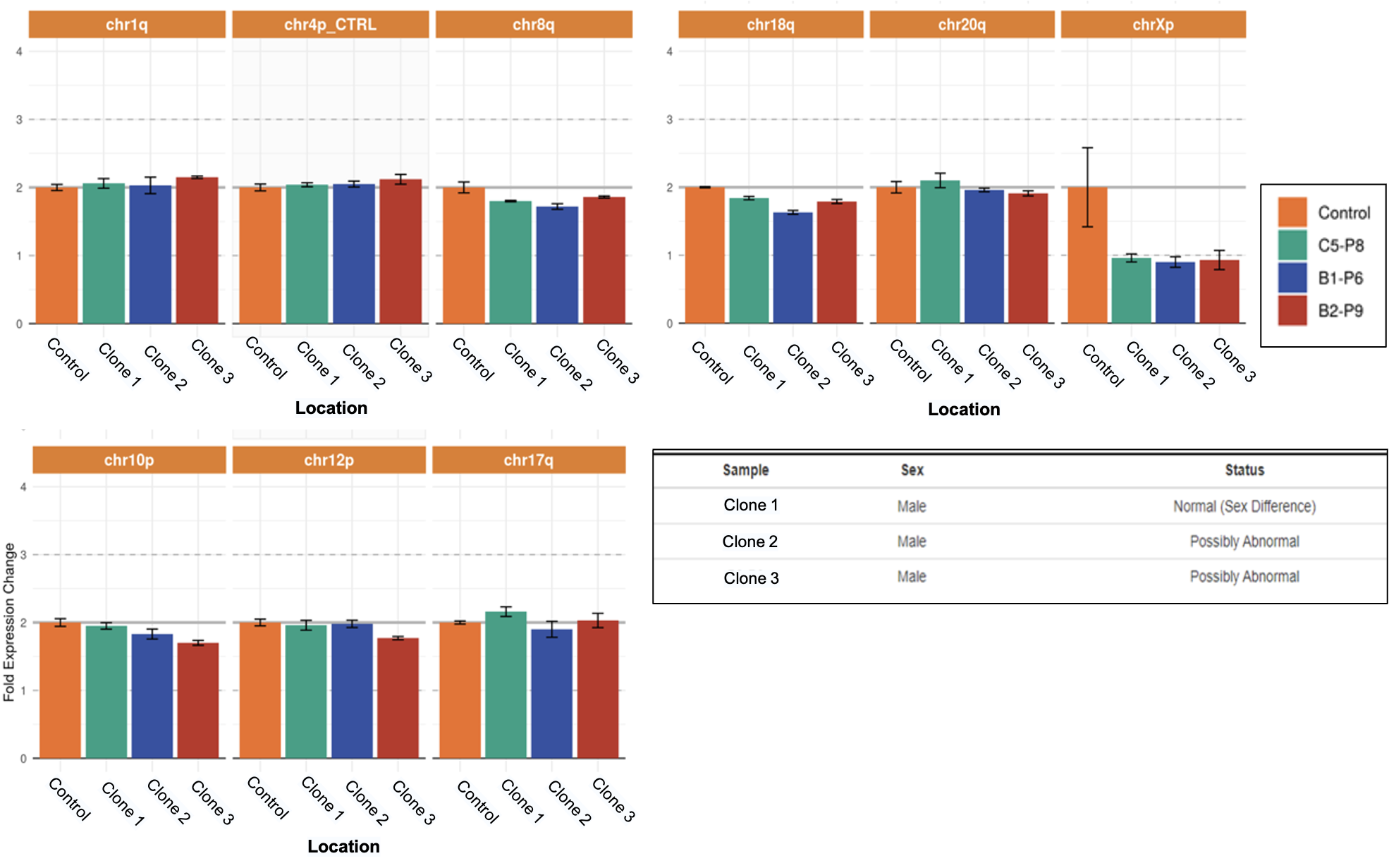
**

**Figure S3. qPCR based karyotype analysis of genomic DNA extracted from different iPSC clones of MLC patient-derived iPSCs.** Clone 1 bears a normal karyotype with only sex difference as a result of a female genomic DNA control with respect to the male cell line tested.

**
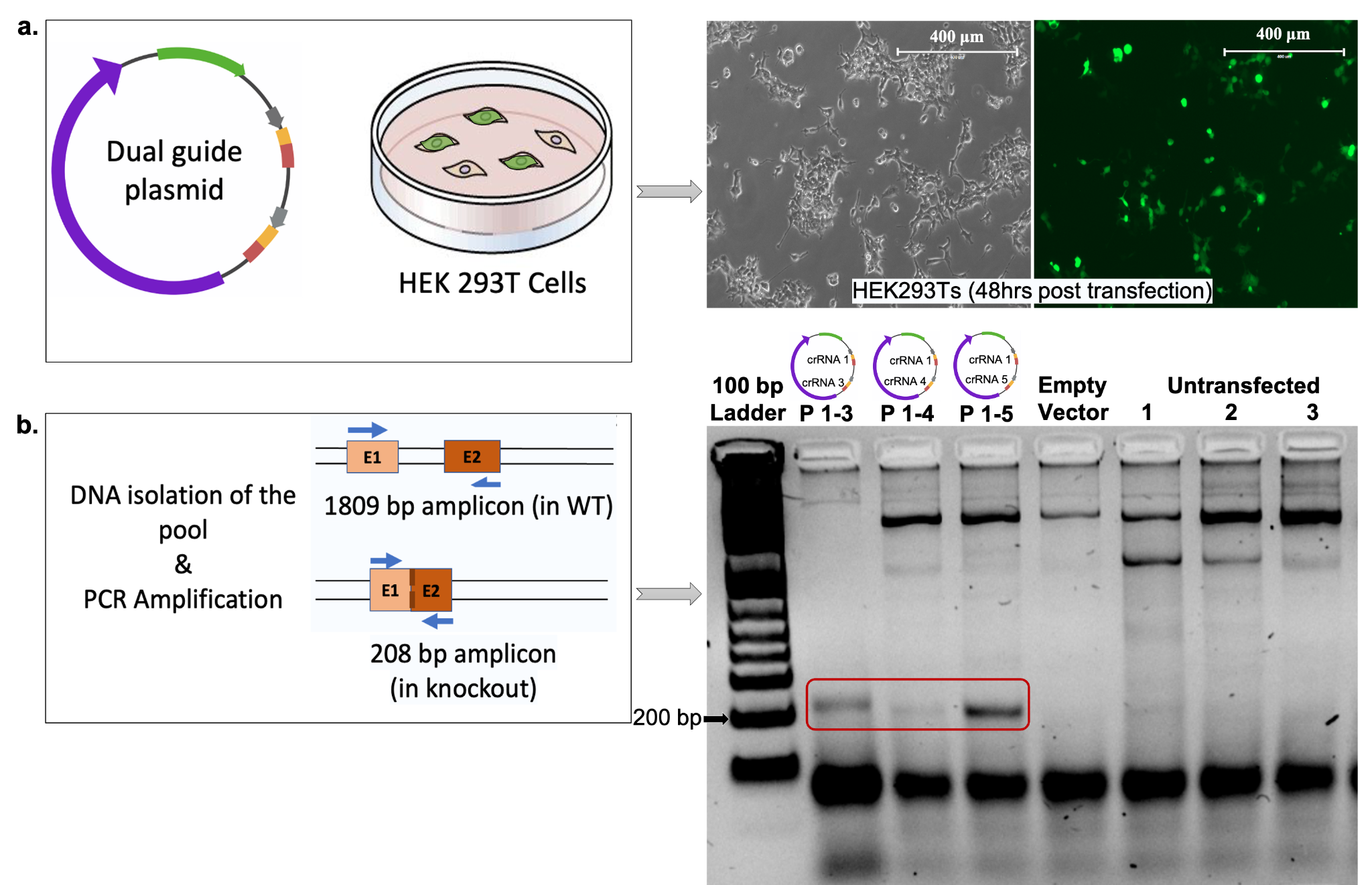
**

**Figure S4. Transfection efficiency and validation of the dual guide plasmid in HEK293T cells.** a. Fluorescence check of the efficiency of genome editing in the pooled cell population. b. Plasmid combination P1-3 and P1-5 resulted in a knockout. P1-3 was taken forward to perform electroporation in human iPSCs following validation via PCR-based screens in HEK cells’ (treated with this dual guide RNA plasmid) genomic DNA.

**
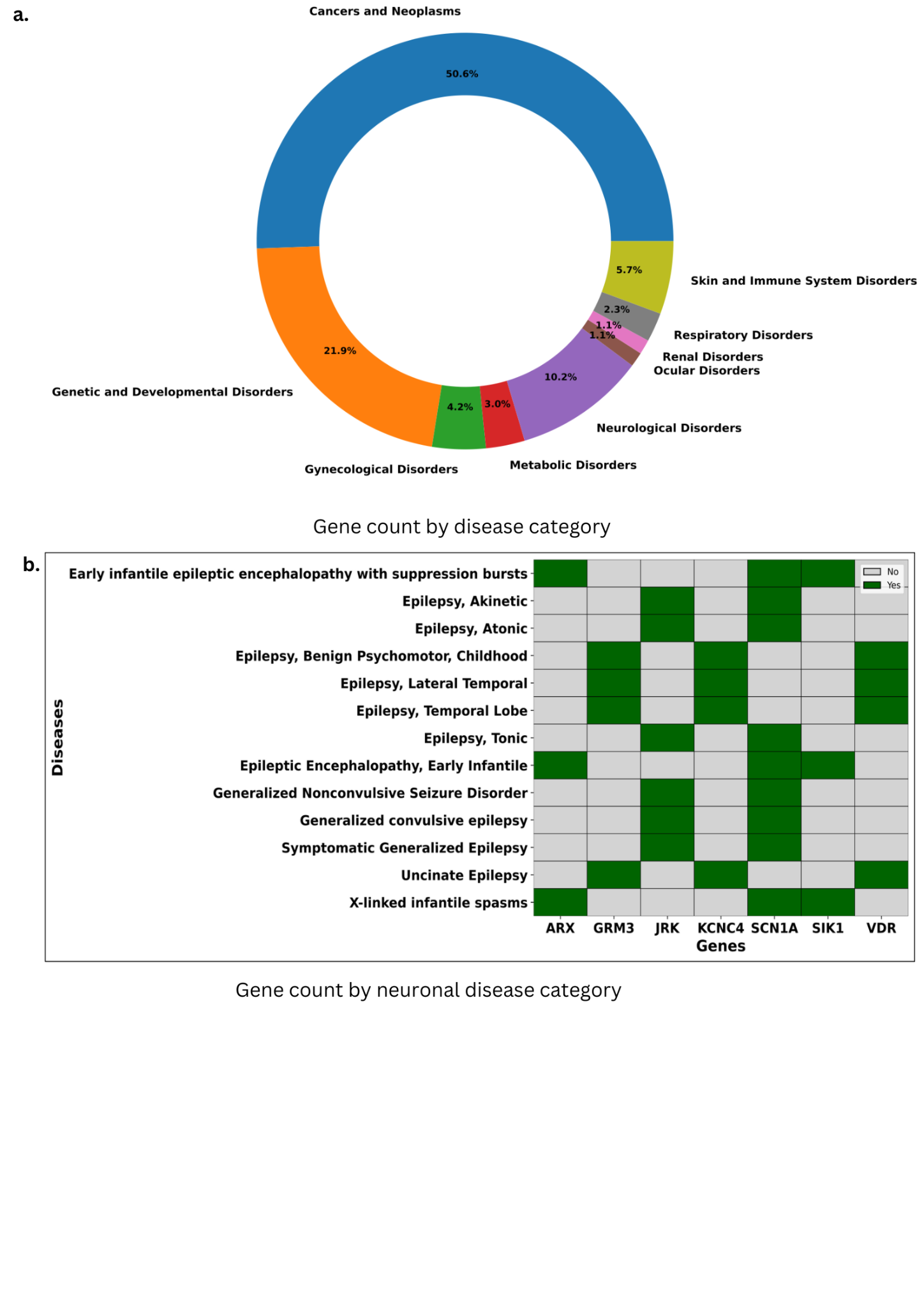
**

**Figure S5. Association of differentially expressed genes in *MLC1* ablated NSCs with human disorders.** a. System-wide disorders associated with MLC1 NSCs. b. Neurological diseases associated with *MLC1* disruption.

**Figure S6. Validation of key epilepsy related genes (one each amongst top 10 highly upregulated and top 10 downregulated ones) using qPCR.** a. The grey solid bars represent qPCR findings for Log2FC whereas the shaded bars are RNASeq Log2FC representation for comparison. The LY6H gene shows an opposite trend of expression in qPCR, whereas ALX1 falls in line with downregulation of gene expression. b. UCSC database reveals the presence of multiple isoforms for the LY6H gene while ALX1 has just one.

**Figure S7. Estimation of size differences in the size of nuclei of *MLC1* knockout astrocytes with respect to Scrambled control astrocytes.** *MLC1* knockout astrocytes looked visibly shrunken in size as compared to scrambled control astrocytes.

**
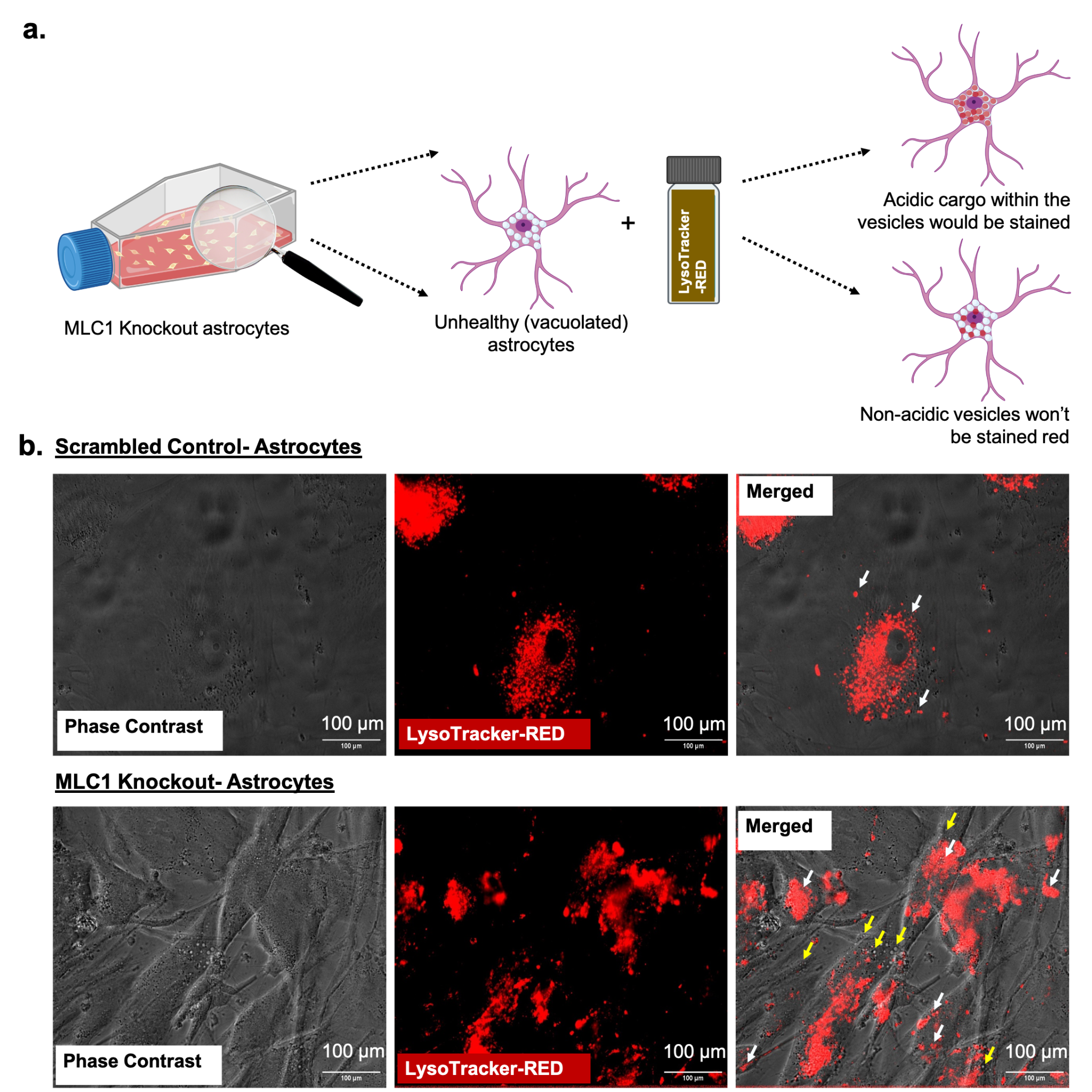
**

**Figure S8. MLC disease-relevant vacuoles are not associated with lysosomes.** a. Astrocytes derived from *MLC1* deleted iPSCs were predicted to develop a vacuolated phenotype representative of MLC disease. b. LysoTracker RED staining was used to observe vacuole acidity. c. LysoTrackerRed did not stain all of the vacuoles observed in the *MLC1* gene knockout astrocytes (yellow arrows indicate unstained vacuoles).
